# Evolution and divergence of SBP-box genes in land plants

**DOI:** 10.1186/s12864-015-1998-y

**Published:** 2015-10-14

**Authors:** Shu-Dong Zhang, Li-Zhen Ling, Ting-Shuang Yi

**Affiliations:** Germplasm Bank of Wild Species, Kunming Institute of Botany of the Chinese Academy of Sciences, Kunming, 650201 China; BGI-Yunnan, BGI-Shenzhen, Kunming, 650106 China

**Keywords:** Divergence, SBP-box gene family, Land plant

## Abstract

**Background:**

*Squamosa* promoter binding protein (SBP)-box family genes encode plant-specific transcription factors that control many important biological functions, including phase transition, inflorescence branching, fruit ripening, and copper homeostasis. Nevertheless, the evolutionary patterns of SBP-box genes and evolutionary forces driving them are still not well understood.

**Methods:**

104 SBP-box gene candidates of five representative land plants were obtained from Phytozome database (v10.3). Phylogenetic combined with gene structure analyses were used to identify SBP-box gene lineages in land plants. Gene copy number and the sequence and structure features were then compared among these different SBP-box lineages. Selection analysis, relative rate tests and expression divergence were finally used to interpret the evolutionary relationships and divergence of SBP-box genes in land plants.

**Results:**

We investigated 104 SBP-box genes from moss, *Arabidopsis*, poplar, rice, and maize. These genes are divided into group I and II, and the latter is further divided into two subgroups (subgroup II-1 and II-2) based on phylogenetic analysis. Interestingly, subgroup II-1 genes have similar sequence and structural features to group I genes, whereas subgroup II-2 genes exhibit intrinsic differences on these features, including high copy numbers and the presence of *miR156*/*miR529* regulation. Further analyses indicate that subgroup II-1 genes are constrained by stronger purifying selection and evolve at a lower substitution rate than II-2 genes, just as group I genes do when compared to II genes. Among subgroup II-2 genes, *miR156* targets evolve more rapidly than *miR529* targets and experience comparatively relaxed purifying selection. These results suggest that group I and subgroup II-1 genes under strong selective constraint are conserved. By contrast, subgroup II-2 genes evolve under relaxed purifying selection and have diversified through gene copy duplications and changes in *miR156/529* regulation, which might contribute to morphological diversifications of land plants.

**Conclusions:**

Our results indicate that different evolutionary rates and selection strengths lead to differing evolutionary patterns in SBP-box genes in land plants, providing a guide for future functional diversity analyses of these genes.

**Electronic supplementary material:**

The online version of this article (doi:10.1186/s12864-015-1998-y) contains supplementary material, which is available to authorized users.

## Background

*Squamosa* promoter binding protein (SBP)-box genes encode transcription factors (TFs) that share a highly conserved DNA-binding domain (the SBP domain) and recognize similar target DNA sequences [1]. This domain consists of approximately 76 amino acid residues and features two zinc-binding sites assembled as Cys-Cys-His-Cys and Cys-Cys-Cys-His, respectively [2]. SBP-box genes are found in green plants from single-celled green algae to multicellular higher plants but not found in prokaryotes, fungi or animals. High-throughput sequencing of plant genomes has identified a number of SBP-box genes at the genomic scale. To date, SBP-box genes of 65 organisms from green algae to flowering plants have been deposited in the Plant Transcription Factor database (PlantTFDB) [3]. These data facilitate a joint phylogenetic analysis of SBP-box genes from algae, bryophytes to angiosperm monocots and eudicots. Based on sequence similarity, green plant SBP-box genes have been divided into two main groups. In phylogenetic analyses, the SBP-box genes of green alga form a monophyletic group and the SBP-box genes of land plants constitute another monophyletic group, albeit with highly diverse subgroups [4]. These results suggest that SBP-box genes predate the origin of land plants, but the SBP-box genes of land plants originated from a common ancestor.

Several studies have indicated that some SBP-box genes in land plants have retained similar functions as SBP-box genes in green alga. In *Chlamydomonas reinhardtii* (green alga), an SBP domain protein *Copper Response Regulator 1* (*CRR1*) binds to the Cu-response element (CuRE) in the promoter region, characterized by the core sequence GTAC [5–7]. This results in the transcriptional activation of copper-deficiency target genes (e.g., *CPX1*) that cause a physiological shift to copper-independent photosynthesis. Similarly, two land plant SBP-box genes, *PpSBP2* and *AtSPL7*, are reported to recognize a GTAC motif of CuREs and regulate nutritional copper signals [8, 9]. These results shed light on the function of SBP-box genes in the common ancestor of land plants. Research on the functions of land plant SBP-box genes during development has focused on loss-of-function phenotypes and their expression patterns. For example, the analysis of three independent transposon-tagged *atspl8* mutants indicated that *AtSPL8* was involved in the regulation of microsporogenesis, megasporogenesis, trichome formation on sepals, and stamen filament elongation [10]. In *Arabidopsis*, *AtSPL3* was involved in floral transition and its constitutive expression caused early flowering [11]. In recent years, *miR156/529* family members have been reported to target land plant SBP-box genes since these miRNA genes originated from land plants [12]. The differential regulation of SBP-box genes by two miRNA families provides an interesting example of the functions that these genes exhibit during land plant development; e.g., the low-level expression of SBP-box genes in an *miR156*-overexpression mutant prolonged the juvenile phase in maize [13] and *Arabidopsis* [14]. These studies suggest that land plant SBP-box genes have diverged and are functionally diverse. Such observations of SBP-box genes in land plants might provide us with useful information for tracing their ancient and divergent evolutionary patterns. Our long-term research questions are: What evolutionary forces drive the divergence of SBP-box genes in land plants? Do these genes evolve under the same constraints and at the same rate?.

Thus, the aim of this study was to assess the evolutionary patterns and dynamics of SBP-box genes in land plants. Phylogenetic analyses, as well as gene structure analyses, were used to identify SBP-box gene lineages in land plants. Gene copy number and sequence and structure characteristics were subsequently compared between groups and subgroups to trace the evolutionary history of these genes in land plants. Finally, selection analysis, relative rate tests and expression divergence were used to interpret the evolutionary relationships and divergence of SBP-box genes in land plants.

## Results

### Phylogenetic analysis of SBP-box genes in land plants

**Fig. 1:**
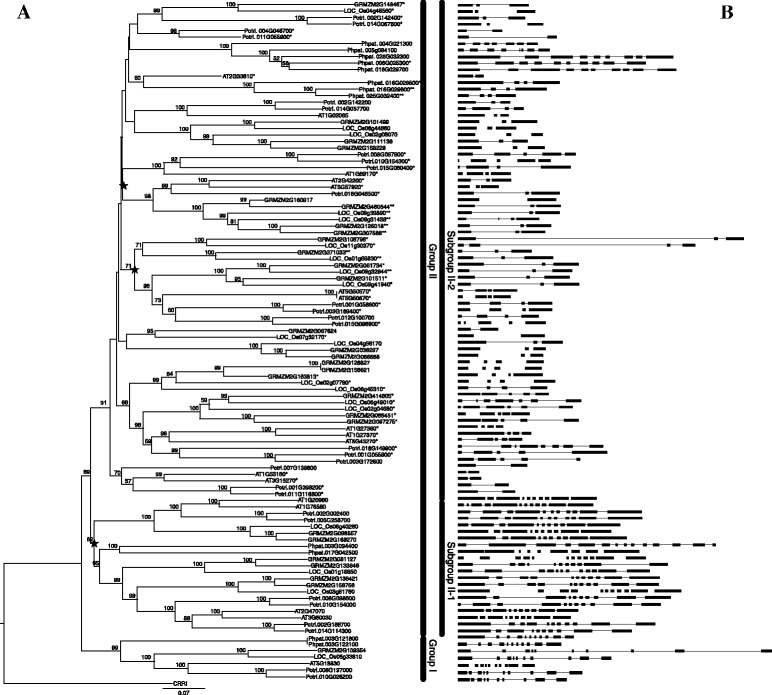
Neighbor-Joining phylogenetic tree of the SBP genes of five land plant species (**a**) and the corresponding gene structure analyses (**b**). Black boxes indicate the exon regions and lines indicate introns. The length of the boxes and lines are scaled based on the length of genes. The number above branch indicates the bootstrap values over 50 %. The genes marked by a single asterisk in the phylogenetic tree are regulated by *miR156* and those marked by double asterisk are cooperatively regulated by both *miR156* and *miR529*. The star indicates the major duplication events

Phylogenetic reconstruction was performed using neighbor-joining (NJ) approaches with JTT and P-distance models based on full-length protein sequences, which resulted in generally similar topologies. In both analyses, the SBP-box genes of land plants formed two lineages (group I and group II) caused by an early duplication event. Group II then gave rise to additional homologs through several rounds of duplication and formed two distinct subgroups (subgroups II-1 and II-2) in the tree (Fig. 1a).

Changes in exon/intron numbers may represent splicing variants has been used to classify genes in many gene families, such as polygalacturonases (PGs) family, MYB family, and bHLH family [15–17]. Therefore, we investigated the exon/intron numbers of 104 SBP-box genes from the groups I, II-1, and II-2. There was a clear relationship between phylogenetic groups and exon numbers (Fig. 1). For example, genes in groups I and II-1 contained no less than 10 exons (Fig. 1b), whereas genes in group II-2 had 2–10 exons. There was also a phylogenetic pattern to the number of exons within genes in group II-2 (Fig. 1b). Therefore, independent evidence from exon/intron numbers supports the group classifications of the SBP-box genes.

### Rapid expansion of group II genes

**Fig. 2:**
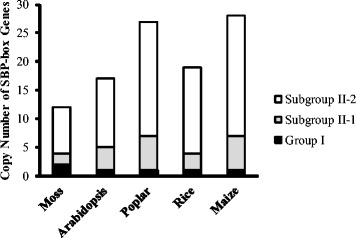
Comparison of the copy number of SBP-box genes in five land plant species. The black, grey, and white boxes represent group I, subgroup II-1, subgroup II-2, respectively

We observed an apparent difference in SBP-box gene copy number among groups in the phylogenetic tree. Of 104 land plant SBP-box genes, only 7 genes belonged to group I and the remaining 97 genes formed group II. The number of group I genes was conserved in the five species studied (Fig. 2): moss had two group I genes and *Arabidopsis*, poplar, rice, and maize had a single group I gene. Variable numbers of group II genes were observed in the five species: moss had 10 group II genes, whereas *Arabidopsis*, poplar, rice, and maize had 16, 26, 18, and 27 genes, respectively. Therefore, there appears to have been an increase in group II SBP-box genes after the divergence of vascular plants. We found that > 65 % of group II gene copies were in subgroup II-2 (Fig. 2). There were comparatively fewer gene copies in subgroup II-1 and the number of genes was relatively conserved among the five species: moss had 2 genes, and *Arabidopsis*, poplar, rice, and maize had 4, 6, 3 and 6 genes, respectively. These comparisons indicate that the expansion of the SBP-box genes in land plants was mainly the result of expansion of group II genes, especially, subgroup II-2 genes in the tracheophytes.

### Sequence and structural characteristics of SBP-box genes

We found that the sequence and structural features of subgroup II-1 SBP-box genes were highly similar to group I genes, although groups II-1 and II-2 were sister in the phylogenetic tree (Fig. 1a). SBP-box genes in groups I and II-1 had no less than 10 exons (Fig. 1b). In contrast, group II-2 genes had 2–10 exons. A MEME analysis revealed extensive conservation in motif architecture (motifs 1, 2, 3, 5, 6, 7, 8, 13, 14) within the SBP-box genes of groups I and II-1 (Additional file 1). Subgroup II-2 SBP-box genes shared no common motifs but the conserved SBP domain with groups I and II-1 genes (Additional file 1). Nevertheless, we found that some of subgroup II-2 SBP-box genes possessed a unique motif (motif 10), which is the responsive element of *miR156* and *miR529* (Additional file 1). This result indicated that only SBP-box genes of subgroup II-2 could be regulated by the two miRNA families. Another feature of group I and II-1 SBP-box genes is that their protein sequences are longer (average length = 968 aa) than subgroup II-2 sequences (average length = 395 aa) (Additional file 2). Moreover, pairwise comparisons of 104 full-length SBP-box protein sequences revealed some notable features. Group I genes showed > 45 % pairwise sequence identity and group II genes showed > 34 % pairwise sequence identity (Additional file 3). In a protein sequence comparison of two subgroups, the higher sequence identities were found among subgroup II-1 genes (more than 40 % sequence identity for subgroup II-1 genes and 35 % sequence identity among subgroup II-2 genes, respectively) (Additional file 3). These comparisons suggest that subgroup II-1 SBP-box genes retained similar evolutionary features to group I genes after they diverged from each other. In contrast, the genes of subgroup II-2, experienced evolutionary changes since their origin via gene duplication.

### Divergence of substitution rates and selective pressures of SBP-box genes

**Fig. 3:**
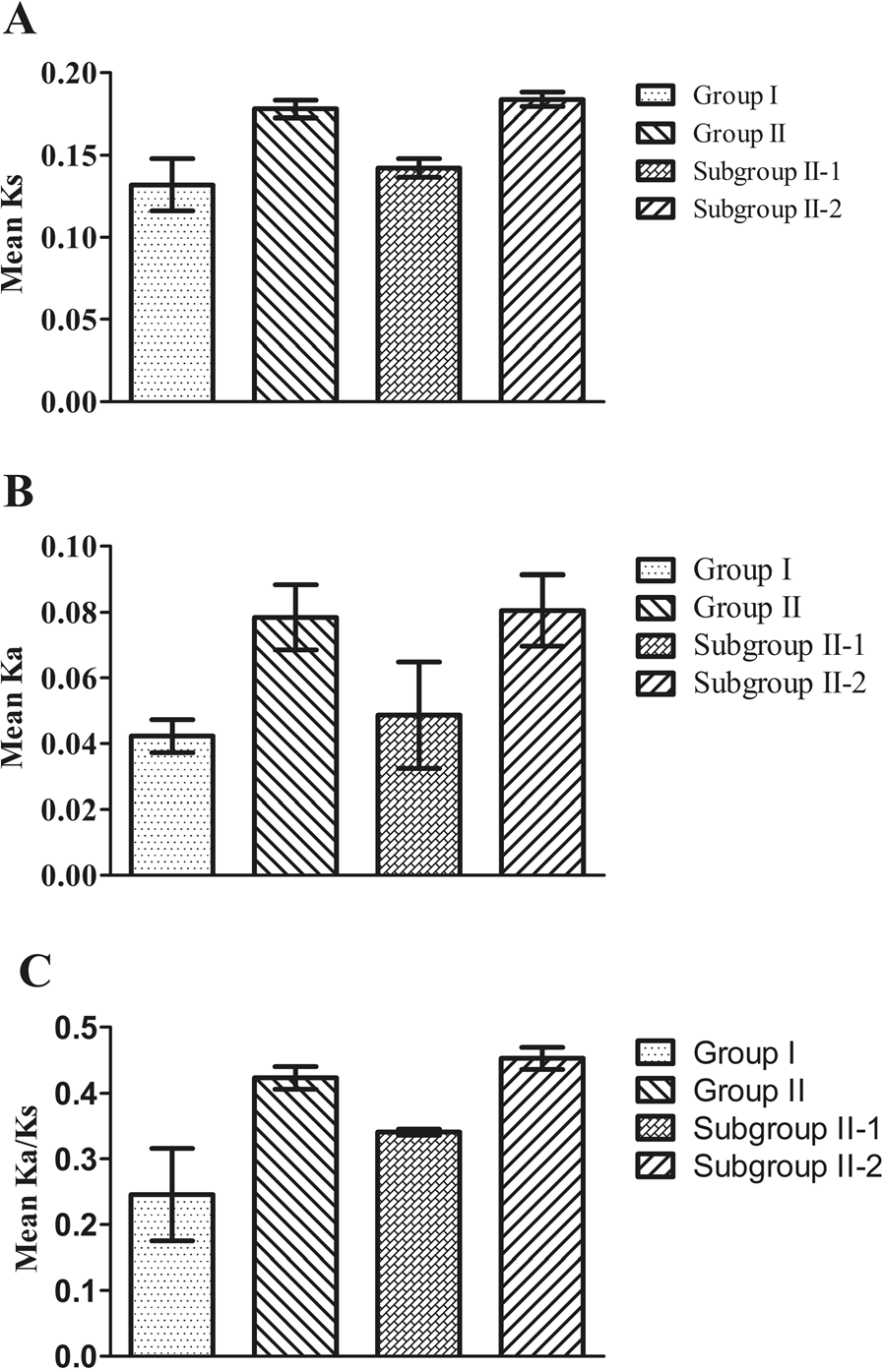
Comparison of the mean Ks value (**a**), the mean Ka value (**b**) and the mean Ka/Ks ratios (**c**) for two groups and subgroups. Error bars indicate the standard error of the mean

The synonymous (Ks) and nonsynonymous substitution rate (Ka) and selection pressure (Ka/Ks) of SBP-box genes were measured for two groups and two subgroups, separately. Our results indicated that the mean Ks and Ka values for group I were lower than those values for group II (Fig. 3a, b). Further analysis revealed that Ka/Ks values were lower than 1.0 (suggesting purifying selection) for both groups. The group I ratio was estimated as 0.24 and group II was estimated as 0.42 (Fig. 3c). We also analyzed the two subgroups and found that subgroup II-1 genes have lower mean Ks and Ka values compared to subgroup II-2 (Fig. 3a, b). In addition, subgroup II-1 genes had a low Ka/Ks ratio (Ka/Ks = 0.34), whereas subgroup II-2 genes had a higher ratio (Ka/Ks = 0.45; Fig. 3c). These results indicated that SBP-box gene copies in different groups experienced different evolutionary rates and selection pressures during evolution. Group I SBP-box genes were subjected to stronger selection pressures and have evolved more slowly than group II genes. A similar difference was found between the slow rates of subgroup II-1 genes in comparison to the faster rates of subgroup II-2 genes.

**Fig. 4:**
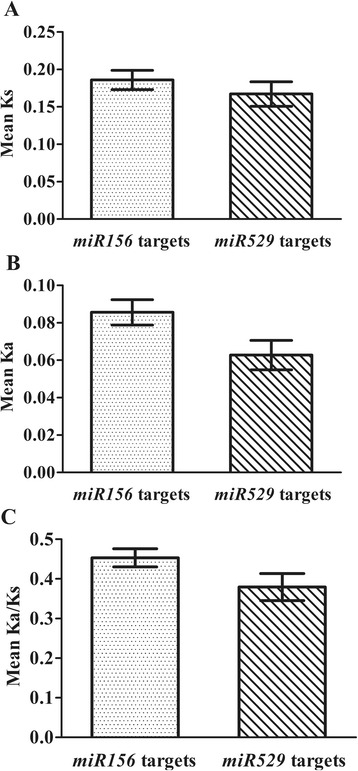
Comparison of the mean Ks value (**a**), the mean Ka value (**b**) and the mean Ka/Ks ratios (**c**) for *miR156* targets and *miR529* targets. Error bars indicate the standard error of the mean

In addition, our prediction suggested that 54 and 11 SBP-box genes in subgroup II-2 were separately targeted by the *miR156* family and *miR156/miR529* families, although not all SBP-box genes of subgroup II-2 were targeted by *miR156* or the *miR529* family. However, we found that 11 SBP-box genes that are cooperatively controlled by *miR156* and *miR529* were in one subset of 54 *miR156* putative targets. For the sake of simplicity, we refer to 11 targets of *miR156* and *miR529* cooperative control as *miR529* targets and we refer to the remaining 43 targets as *miR156* targets. The evolutionary parameters (Ka, Ks, and Ka/Ks) were also estimated between two target datasets. Our results indicated that *miR156* targets had higher mean Ka and Ks values as compared to *miR529* targets (Fig. 4a and b). Meanwhile, a strong difference between Ka/Ks ratios for these two targets was also observed in Fig. 3. The *miR156* targets had elevated Ka/Ks ratios, whereas *miR529* targets had lower Ka/Ks ratios (Fig. 4c). These results indicate that different target genes in subgroup II-2 also exhibited different evolutionary rates and selection pressures.

### Expression divergence of SBP-box genes in *Arabidopsis* and rice

We obtained the expression data of SBP-box genes in *Arabidopsis* and rice from their respective genome annotation databases (see Methods). In these two organisms, the expression patterns of all SBP-box genes were examined in five major tissues: leaf, shoot, petiole, cotyledon and hypocotyl in *Arabidopsis* and leaf, shoot, seed, endosperm and anther in rice. Group I and subgroup II-1 SBP-box genes are ubiquitously expressed in all tissues of *Arabidopsis* and rice (Table 1). On the other hand, subgroup II-2 SBP-box genes have tissue-specific expression patterns. For example, AT1G27360 and LOC_Os09g32944 from subgroup II-2 were only detected in the shoots of *Arabidopsis* and rice (Table 1). These results demonstrate that group I and subgroup II-1 SBP-box genes are ubiquitously expressed, whereas subgroup II-2 SBP-box genes have restricted expression patterns.

**Table 1:** The expressional distribution of SBP-box genes in *Arabidopsis* and rice

Organism	Class	Gene_ID	Leaf	Shoot	Petiole	Cotyledon	Hypocotyl
*Arabidopsis*	I	AT5G18830	+	+	+	+	+
	II-1	AT1G20980	+	+	+	+	+
	II-1	AT1G76580	+	+	+	+	+
	II-1	AT2G47070	+	+	+	+	+
	II-1	AT3G60030	+	+	+	+	+
	II-2	AT1G27360		+	+		
	II-2	AT1G53160	+	+	+	+	+
	II-2	AT1G69170					
	II-2	AT2G33810	+		+	+	
	II-2	AT2G42200	+	+	+		
	II-2	AT3G15270					
	II-2	AT3G57920		+			
	II-2	AT5G43270	+	+	+	+	+
	II-2	AT5G50570					
	II-2	AT5G50670					
	II-2	AT1G02065					
	II-2	AT1G27370	+	+	+	+	+
Organism	Class	Gene_ID	Leaf	Shoot	Seed	Endosperm	Anther
Rice	I	LOC_Os05g33810	+	+	+	+	+
	II-1	LOC_Os01g18850	+	+	+	+	+
	II-1	LOC_Os03g61760	+	+	+	+	+
	II-1	LOC_Os08g40260	+	+	+	+	+
	II-2	LOC_Os01g69830	+	+			+
	II-2	LOC_Os08g39890	+	+	+	+	+
	II-2	LOC_Os09g31438		+			+
	II-2	LOC_Os09g32944		+			
	II-2	LOC_Os02g04680	+	+	+	+	+
	II-2	LOC_Os02g07780	+	+	+	+	+
	II-2	LOC_Os04g46580					
	II-2	LOC_Os06g45310	+	+	+	+	+
	II-2	LOC_Os06g49010	+	+	+	+	+
	II-2	LOC_Os07g32170	+	+			+
	II-2	LOC_Os08g41940					+
	II-2	LOC_Os11g30370	+	+			+
	II-2	LOC_Os04g56170	+	+			
	II-2	LOC_Os06g44860					
	II-2	LOC_Os02g08070					

## Discussion

The SBP-box genes constitute a family of plant-specific transcription factors and are found in algae and land plants [18]. Several studies have suggested that land plant SBP-box genes share a common origin with algae SBP-box genes [4, 18]. Land plants develop complex organs, such as stems, leaves, and reproductive structures that are not necessarily present in algae. In most cases, land plant SBP-box genes have been shown to play important roles in the general development of plant structures [19]. It has been reported that land plant SBP-box genes are involved in the transitions from juvenile to adult phases and from vegetative to reproductive phases. They are also involved in trichome development, apical dominance, inflorescence branching, fruit ripening, plastochron length and pollen sac development (see the detailed review of SBP-box gene functions in Preston and Hileman (2013)). Therefore, SBP-box gene functions diversified in land plants after their divergence from their common ancestor with algae. Their functional diversity might be correlated with the differentiation of developmental characteristics in land plants. Previous studies have suggested that land plant SBP-box genes retain their capacity to perform ancient functions regulating copper homeostasis, just as they do in algae [8, 9]. Therefore, SBP-box genes in land plants might present ancient and neo-functional evolutionary patterns. Phylogenetic analyses of SBP-box genes are useful guides for studying gene roles and gene family diversification. During plant evolution, genes with similar functions in different species are generally closely related and form a clade in a phylogenetic analysis. We found that the 104 SBP-box genes from moss, *Arabidopsis*, poplar, rice, and maize form two groups (group I and II). This result is consistent with previous work on land plant SBP-box genes [4]. However, our phylogenetic reconstruction based on full-length proteins also divided group II genes into two distinct subgroups, rather than the seven subgroups found when domain sequences were analyzed (subgroup II a-g) [4]. Compared to full-length proteins, domain sequences indicate low support (<50 % bootstrap) for relationships among lineages of group II genes. One possible reason for this low support is the limited number of informative characters within the alignable SBP-box domain sequences. Thus, full-length proteins used in this study provide more informative characters and relatively strong support for SBP-box gene lineages. Nevertheless, the distinct evolutionary patterns of land plant SBP-box gene lineages and the nature of the evolutionary forces driving them are still not clearly understood.

Our results indicate that group I SBP-box genes with a low evolutionary rate exhibit a conserved evolutionary pattern that is under strong purifying selection. They have characteristics of conserved genes: long protein sequences, a complex gene structure, lack of *miR156/529* binding sites and nearly ubiquitous expression across different organs and tissues in distantly related plant species (Fig. 1, Table 1, and Additional file 1 and 2). By comparison, group II genes have diverged into two subgroups (subgroup II-1 and II-2) under relaxed selection pressures (Fig. 1). In our results, subgroup II-1 genes are sister to subgroup II-2 genes and there should be more similarities between these two subgroups. However, our results show that subgroup II-1 genes have more similar sequence and structural features to group I genes than to subgroup II-2 genes (Fig. 1and Additional file 1 and 3). Furthermore, the function of subgroup II-1 genes are more similar to the functions of group I genes. For example, subgroup II-1 gene *PpSBP2* functions in copper homeostasis, just as group I gene *AtSPL7*, and their distant paralog, *CRR1* (included as an outgroup) [6, 8, 9]. Therefore, we hypothesize that subgroup II-1 genes might have a conserved evolutionary pattern, just as group I genes, whereas subgroup II-2 genes exhibit divergent patterns during evolution. Estimation of evolutionary parameters (Ka, Ks, Ka/Ks) support our hypothesis. Subgroup II-1 genes have a slower evolutionary rate and are under strong purifying selection in comparison to subgroup II-2 genes. This contributes to their conserved pattern (Fig. 3a, b and c). Usually, slower sequence mutation results in higher sequence conservation and faster sequence mutation results in lower sequence conservation. Our results indicate that sequence conservation, characterized by sequence identity, among SBP-box gene groups is correlated with their evolutionary rates, supporting the idea that these groups experienced a different evolutionary scenario under different selection strengths (Additional file 3). Meanwhile, another indicator of evolutionary rate is the expression patterns of different groups. In this case, expression pattern provides further support for our hypotheses because ubiquitously expressed genes evolve more slowly than tissue-specific genes (Table 1). Based on our analyses, we conclude that group I and subgroup II-1 genes have retained an ancestral functions and conserved expression patterns. Additionally, sequence, structure, and expression differences between subgroup II-2 genes and group I and subgroup II-1 genes indicate that subgroup II-2 genes have evolved under relaxed purifying selection and have apparently divergent evolutionary patterns. Previous studies have revealed that genes with rapid evolutionary rates under relaxed purifying selection may more readily adopt new forms of biased expression during the evolution of alternate phenotypes [20]. In particular, there are various aspect of development in the leaves, flowers and fruits that are controlled by different subgroup II-2 genes (reviewed in Preston and Hileman (2013)). Therefore, we infer that the large number of genes in subgroup II-2 might be closely related to the diversification of development characteristics of land plants by altering their sequence and expression patterns.

Gene duplication is thought to provide the raw genetic resources for natural selection to act on. We suggested in a previous study that segmental and tandem gene duplications predominated during the expansion of the SBP-box gene family [4]. Our results here demonstrate that the genes of SBP-box groups and subgroups exhibit different patterns in copy number after they were derived from independent gene duplication events. The genes of group I and subgroup II-1 demonstrate highly and moderately conserved copy number, respectively, whereas subgroup II-2 genes exhibit a variable number of copies and underwent a rapid diversification, especially in tracheophytes (Fig. 2). In general, the duplicated genes with fewer copies evolve significantly more slowly than those with many copies [21]. Therefore, our results provide a third line of evidence for the evolutionary rates of different gene groups in addition to evidence from sequence conservation and expression patterns. Why did this rapid expansion of subgroup II-2 genes occur in tracheophytes? It is well known that organ structures in tracheophytes are highly diverse in form and size. One possible explanation is that the expansion of subgroup II-2 genes might have facilitated the morphological diversification of tracheophytes through neofunctionalization and impacts on developmental processes. Previous studies have indicated that miRNA-mediated regulations have also contributed to the phenotypic diversification of plants, accompanied by rapid expansion in early land plant evolution [22]. We found that *miR156/miR529* binding sites were present in subgroup II-2 SBP-box genes, but were not present in group I or subgroup II-1 genes (Additional file 1 and 4). Although we did not find evidence that *miR156* and *miR529* target all subgroup II-2 genes, the number of SBP-box genes targeted by *miR156* is almost five times that of the number of genes cooperatively targeted by *miR156* and *miR529* (54 vs. 11, Additional file 4). Our previous work revealed that SBP-box genes targeted by *miR156* evolve more rapidly and experience more relaxed purifying selection than genes targeted by both *miR156* and *miR529* [12]. A similar result was also obtained with the different datasets in this present study (Fig. 4a, b and c). Therefore, we inferred that relaxed purifying selection might allow mutation at the *miR156* binding sites and produce greater sequence diversity, which contributes to the increasing number of *miR156* target genes. Furthermore, one of our recent studies revealed that the *miR156* family continually duplicates its gene copies, but retains conserved mature sequences, which would harmonize the regulation of increasing numbers of *miR156* targets [23]. In contrast, the fast mutation rate of *miR529* and its high gene loss rate are two major modes of inactivation of *miR529* family members [23]. Our previous results, together with evidence for a strong selective constraint against variations in binding sites cooperatively controlled by *miR156* and *miR529*, provides evidence that the contraction of *miR529* family members might lead to fewer SBP-box genes regulated by a combination of *miR156*/*miR529*. For example, none of SBP-box genes were cooperatively regulated by *miR156* and *miR529* because there are no *miR529* candidates found in core eudicots (i.e. *Arabidopsis* and poplar). However, potential SBP-box targets controlled by *miR156* and *miR529* in these species were predicted when the *miR529* genes from rice, maize and moss were used [23]. Taken together, the rapid expansion of subgroup II-2 genes and regulatory changes of *miR156/529* on these genes could serve as new sources of functional diversity and confer phenotypic differences during development. However, the sampling in our present study only reflects the division of SBP-box genes between bryophytes and tracheophytes and information on SBP-box genes in ferns and gymnosperms is still unknown. Therefore, further investigations will need to gather data from a broader taxonomic sampling and reveal the differences of SBP-box genes at a finer evolutionary scale.

## Conclusions

Land plant SBP-box genes are divided into group I and II, and the latter is further divided into two subgroups (subgroup II-1 and II-2) through several round of duplication. Group I genes and subgroup II-1 genes under strong purifying selection evolve at a low substitution rate and share the conserved evolutionary features. By contrast, subgroup II-2 genes experiencing comparatively relaxed purifying selection evolve more rapidly and have continually diversified through gene copy duplications and changes in *miR156/529* regulation, which contributes to the morphological diversifications in land plants. Such study will provide better insights into understanding evolutionary divergence of the SBP-box genes in land plants and provide a guide for future functional diversity analyses of these genes.

## Methods

### Collection of SBP-box gene sequences in land plants

The SBP-box gene candidates were first obtained from gene prediction sets provided by the comparative genome database Phytozome v10 [24]. Their protein sequences were then collected from five land plant species: one moss (*Physcomitrella patens*), two eudicots (*Arabidopsis thaliana* and poplar, *Populus trichocarpa*) and two monocots (rice, *Oryza sativa* subsp. *japonica* and maize, *Zea mays*) (Additional file 2). Although these five species have well-annotated genomes, SBP-box gene candidates might be mis-annotated during the automated genome annotation process. Hence, these candidates were first analyzed using SMART to confirm the presence of an SBP domain in the protein sequence [25]. Twelve protein fragments containing partial SBP domains were excluded from subsequent analyses because of the possibility that they were pseudogenes. Finally, a total of 104 SBP-box genes were selected from five representative species of land plants. The corresponding domain and mRNA sequences were also downloaded from Phytozome v10 [24]. The SBP-box genes used in this study are summarized in Additional file 2.

### Sequence alignment and phylogenetic reconstruction

We aligned full-length SBP-box protein sequences using Clustal X [26] and manually trimmed the edges of the alignment and discarded the excessive gaps. Phylogenetic relationships were separately reconstructed using the neighbor-joining (NJ) method in MEGA vers. 6 [27] with the Jones-Taylor-Thornton (JTT) model and P-distance model. Bootstrap values were calculated with 1000 replicates in MEGA. In addition, the outgroup sequence (*CRR1*) was chosen from green algae homologs based on our previous analysis [4]. All phylogenetic data has been deposited in TreeBASE (Study ID S18242).

### Gene structure and motif analyses

Exon/intron sites and length data were extracted based on five respective genome annotation GFF files from Phytozome v10.3. A diagram of exon/intron structures was created using the online Gene Structure Display Server (GSDS, http://gsds.cbi.pku.edu.cn/). Motifs were detected using MEME version 4.9.1 with the parameters described in our previous study [28].

### Prediction of *miR156/miR529* target genes

Apart from well-annotated genomes, these five species also have comprehensive miRNA information, in which *miR156* and *miR529* genes had been completely annotated using deep sequencing data. All mature sequences of *miR156* and *miR529* genes were downloaded from miRBase release 21 [29]. Binding sites on SBP-box gene transcripts were identified in these five land plants by using the online psRNATarget server (http://plantgrn.noble.org/psRNATarget/) with default settings [30]. To further increase the stringency of prediction, we used empirical parameters as a second filter [31] as described in our previous study [12]. Finally, our analyses led to the prediction of 54 SBP-box genes as the putative targets for *miR156* and 11 SBP-box genes as the putative targets for *miR529* (Fig. 1 and Additional file 4).

### Estimation of selection and substitution rates

The sequence alignment and a NJ tree (P-distance model) were used to calculate nonsynonymous (Ka) and synonymous (Ks) substitution rates and their ratio (Ka/Ks) for each group/subgroup branch through a Ka/Ks online tool (http://services.cbu.uib.no/tools/kaks). The ratio of Ka/Ks provides a sensitive test of natural selection. A statistically significant Ka/Ks ratio lower than, equal to, or greater than 1.0 can indicate purifying selection, neutral evolution and positive selection, respectively.

### Expression analyses of SBP-box genes in *Arabidopsis* and rice

To investigate the expression patterns of SBP-box genes, we collected available expression data from species genome annotation databases. Information on expression of each SBP-box gene in *Arabidopsis* was obtained from TAIR version 10 (https://www.arabidopsis.org/) and expression data for rice was downloaded from TIGR version 7 (http://rice.plantbiology.msu.edu/). If the expression level of a gene is more than zero in tissues, this gene is considered to be expressed in these tissues. Or else, the gene is considered to be not expressed in tissues when its expression level is equal to zero.

## Additional files

Additional file 1:
**Schematic diagram of motif architectures of every group or subgroup.** (PDF 634 kb) Additional file 2:
**Summary of information on SBP-box genes from five land plant species: moss,** ***Arabidopsis*** **, poplar, rice and maize used in this study.** (XLS 20 kb) Additional file 3:
**Sequence identity of full-length SBP-box proteins in each diverged group and subgroups. Error bars indicate the standard error of the mean.** (PDF 1824 kb) Additional file 4:
**The detail information of predicted targets for** ***miR156*** **and** ***miR529*** **in five land plants used in this study.** (XLS 37 kb)

## Abbreviations

SBP: *Squamosa* promoter binding protein
TFs: Transcription factors
PlantTFDB: Plant Transcription Factor database
*CRR1*: *Copper Response Regulator 1*
NJ: Neighbor-joining
JTT: Jones-Taylor-Thornton
PGs: Polygalacturonases
Ks: synonymous substitution rate
Ka: nonsynonymous substitution rate
Ka/Ks: the nonsynonymous to synonymous substitution rate ratio
GSDS: Gene Structure Display Server
