# Supplementary figures and images for: Evolution and divergence of SBP-box genes in land plants
Source: BMC Genomics. 2015 Oct 14;16:787. doi: 10.1186/s12864-015-1998-y (PMC4606839; doi:10.1186/s12864-015-1998-y)

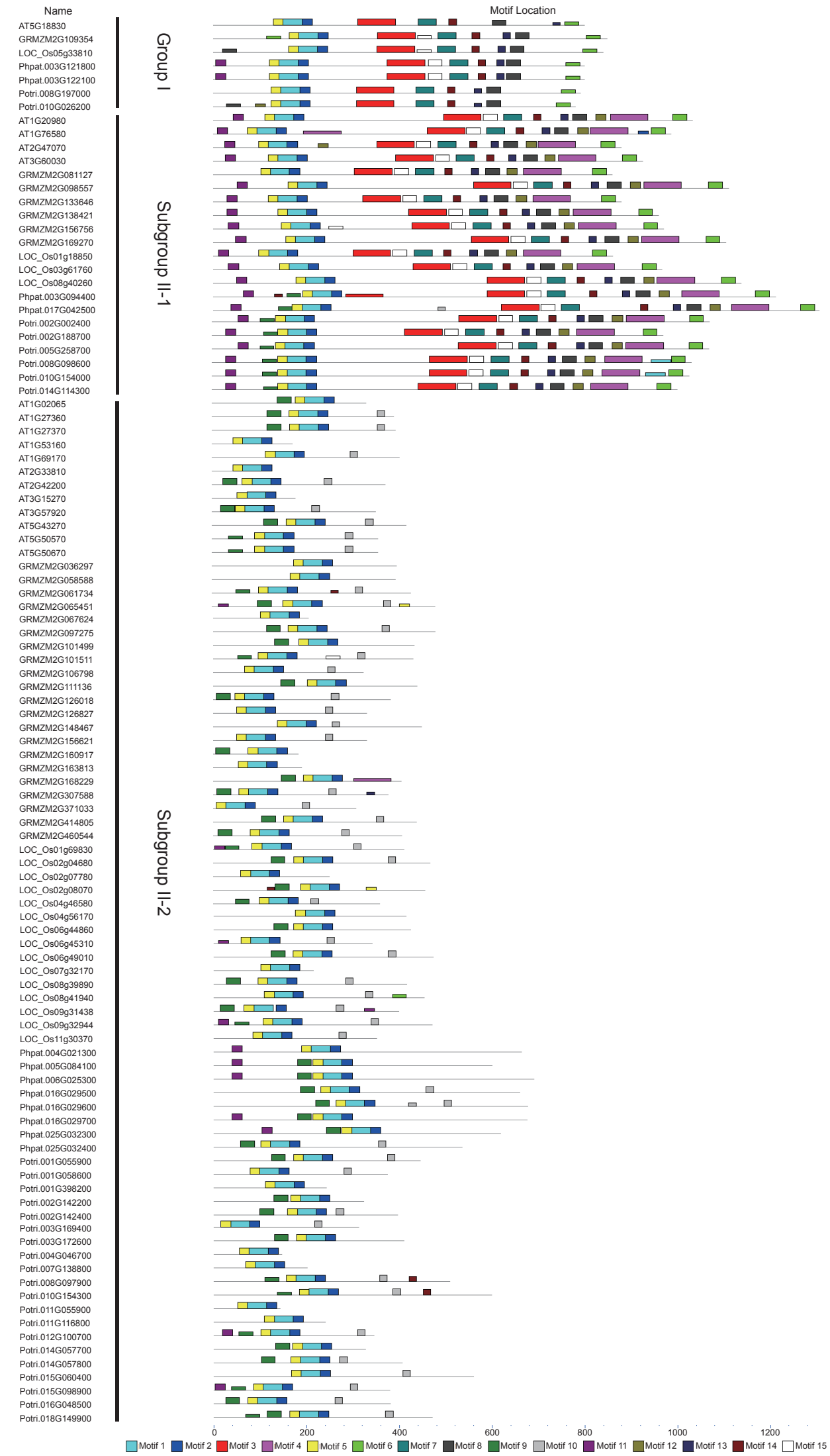

Supplement: Additional file 1: — Schematic diagram of motif architectures of every group or subgroup. (PDF 634 kb) [file 12864_2015_1998_MOESM1_ESM.pdf]

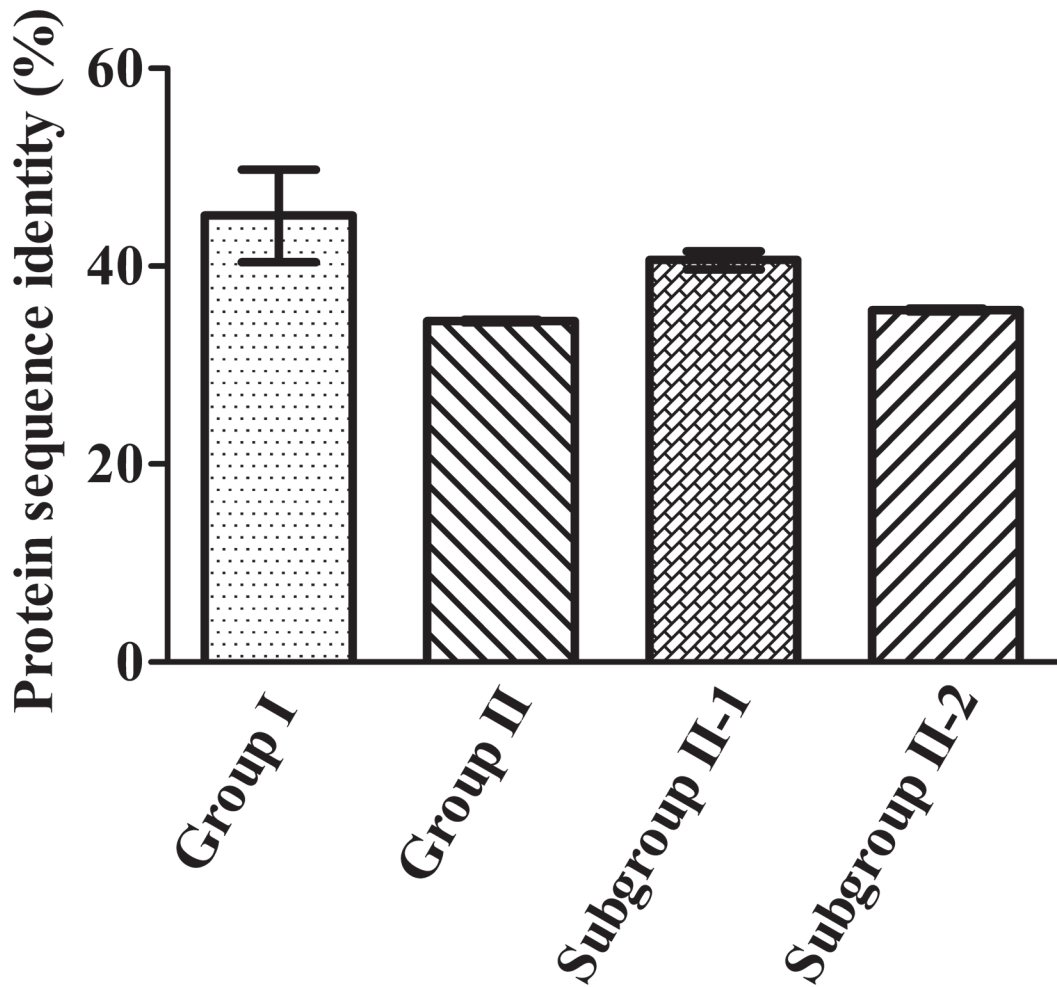

Supplement: Additional file 3: — Sequence identity of full-length SBP-box proteins in each diverged group and subgroups. Error bars indicate the standard error of the mean. (PDF 1824 kb) [file 12864_2015_1998_MOESM3_ESM.pdf]
